# Mice lacking the PSD-95–interacting E3 ligase, Dorfin/Rnf19a, display reduced adult neurogenesis, enhanced long-term potentiation, and impaired contextual fear conditioning

**DOI:** 10.1038/srep16410

**Published:** 2015-11-10

**Authors:** Hanwool Park, Jinhee Yang, Ryunhee Kim, Yan Li, Yeunkum Lee, Chungwoo Lee, Jongil Park, Dongmin Lee, Hyun Kim, Eunjoon Kim

**Affiliations:** 1 Graduate School of Medical Science and Engineering, Korea Advanced Institute of Science and Technology (KAIST), Daejeon 305-701, Korea; 2 Department of Biological Sciences, KAIST, Daejeon 305-701, Korea; 3 Center for Synaptic Brain Dysfunctions, Institute for Basic Science (IBS), Daejeon 305-701, Korea; 4 Department of Anatomy and Division of Brain Korea 21. Biomedical Science, College of Medicine, Korea University, Seoul 136-704, Korea

## Abstract

Protein ubiquitination has a significant influence on diverse aspects of neuronal development and function. Dorfin, also known as Rnf19a, is a RING finger E3 ubiquitin ligase implicated in amyotrophic lateral sclerosis and Parkinson’s disease, but its *in vivo* functions have not been explored. We report here that Dorfin is a novel binding partner of the excitatory postsynaptic scaffolding protein PSD-95. Dorfin-mutant (*Dorfin*^−/−^) mice show reduced adult neurogenesis and enhanced long-term potentiation in the hippocampal dentate gyrus, but normal long-term potentiation in the CA1 region. Behaviorally, *Dorfin*^−/−^ mice show impaired contextual fear conditioning, but normal levels of cued fear conditioning, fear extinction, spatial learning and memory, object recognition memory, spatial working memory, and pattern separation. Using a proteomic approach, we also identify a number of proteins whose ubiquitination levels are decreased in the *Dorfin*^−/−^ brain. These results suggest that Dorfin may regulate adult neurogenesis, synaptic plasticity, and contextual fear memory.

Protein ubiquitination regulates diverse aspects of neuronal development and function. E3 ubiquitin ligases, which number in the hundreds (~400–500 in mice and humans), are key components of the protein ubiquitination system, playing important roles in determining substrate specificity. Protein ubiquitination is known to lead to protein degradation by the 26S proteasome; however, accumulating evidence indicates that it also serves additional functions, such as regulation of protein function, trafficking and subcellular localization, as well as protein-protein interactions.

Protein ubiquitination in the nervous system regulates different stages of neuronal development, including neurogenesis, migration, neuritogenesis, and synaptogenesis^1^,^2^,^3^,^4^,^5^. In addition, ubiquitination of synaptic proteins is thought to regulate diverse aspects of synaptic structure, function, and plasticity. Known substrates of synaptic ubiquitination include scaffolding/adaptor proteins, receptors, and signaling molecules. Specific examples and related E3 ligases include PSD-95–Mdm2^6^, GKAP/SAPAP–Trim3^7^,^8^, Shank/ProSAP^7^, SPAR–βTRCP^9^,^10^, AKAP79/150^7^, Homer-1a^11^, CaMKIIα^12^, liprin-α1–APC/C^13^,^14^, ephexin-5–Ube3A^15^, Arc–Ube3a/Triad3a^16^,^17^, AMPA receptors (AMPARs)–Nedd4-1/RNF167/APC/C^14^,^18^,^19^,^20^,^21^,^22^, NMDA receptors (NMDARs)–Mib2^23^, mGluR1α–Siah1A^24^,^25^, mGluR5–Siah1A^25^, GABA_A_ receptors (γ2 subunit)^26^,^27^, Munc13-1–Fbxo45^28^, RIM1–SCRAPPER^29^, and Piccolo/Bassoon–Siah1A^30^.

Dorfin, a RING finger E3 ubiquitin ligase, was originally identified as a component of the XY body of spermatocytes and centrosomes^31^. Dorfin (840 amino acids long in mice) contains two RING domains flanking the in-between ring (IBR) domain, and two transmembrane domains, although the precise membrane topology of the protein is not well established. Previous studies have implicated Dorfin in familial amyotrophic lateral sclerosis (ALS) and Parkinson’s disease^32^,^33^,^34^,^35^,^36^,^37^,^38^,^39^,^40^. In support of a neuroprotective role for Dorfin in ALS, Dorfin ubiquitinates ALS-associated mutant SOD1 (superoxide dismutase 1) protein^33^, and, when overexpressed in a mouse model of familial ALS, reduces the amount of mutant SOD1 proteins and suppresses neurological phenotypes and motor neuron death^40^. However, little is known about the functions of Dorfin in the normal brain, including its protein-protein interactions, substrate proteins, and functional consequences of substrate protein ubiquitination. In addition, *in vivo* functions of Dorfin have not been explored using gene-knockout approaches.

In the present study, we found that Dorfin interacts with the abundant excitatory postsynaptic scaffolding protein PSD-95. *Dorfin*^−/−^ mice show suppressed adult neurogenesis and enhanced long-term potentiation (LTP) in the dentate gyrus (DG) and impaired contextual fear conditioning, suggesting that Dorfin is important for adult neurogenesis, synaptic plasticity, and learning and memory.

## Results

### Dorfin interacts with PSD-95

**Figure 1:**
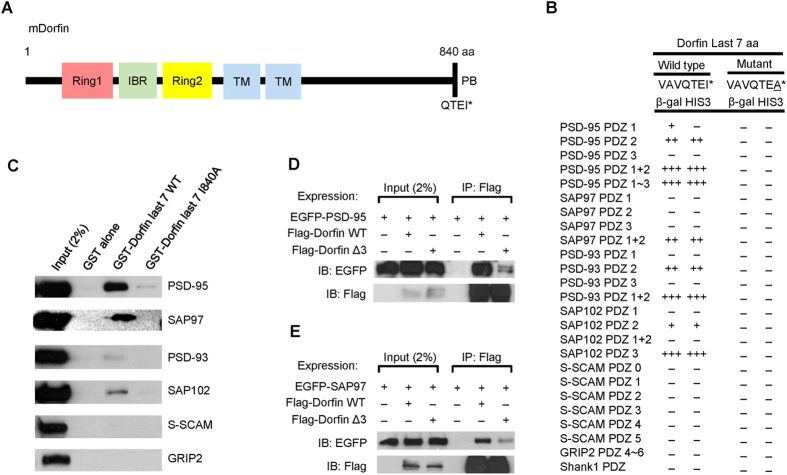
Dorfin interacts with PSD-95 family proteins in yeast two-hybrid, GST pull-down, and coimmunoprecipitation assays. (**A**) Domain structure of mouse Dorfin. IBR, In-between RING; TM, transmembrane domain; PB, PDZ domain-binding motif. (**B**) Yeast two-hybrid interactions of Dorfin with PSD-95. PDZ domains from PSD-95 family proteins, S-SCAM and other PDZ proteins in pGAD10 (prey vector) were tested for binding to Dorfin (aa 834–840; wild type and the I840A mutant, in which the last Ile residue was changed to Ala) in pBHA (bait vector) in yeast two-hybrid assays. β-Galactosidase (β-Gal) activity: +++, <45 mins; ++, 45–90 mins; +, 90–240 mins; −, no significant β-Gal activity. HIS3 activity: +++, >60%; ++, 30–60%;, +, 10–30%; −, no significant growth. (**C**) Pull down of full-length PSD-95 family proteins and control PDZ proteins (S-SCAM and GRIP2) by GST-Dorfin fusion proteins. GST-Dorfin (aa 834–840; WT and I840A), or GST alone, was used to bring down the indicated proteins expressed in HEK293T cells, followed by immunoblotting. (**D**,**E**) Coimmunoprecipitation of Dorfin with PSD-95 or SAP97. HEK293T cells doubly transfected with Flag-Dorfin (full length; WT or Δ3 missing the last three aa residues) and PSD-95, or SAP97, were immunoprecipitated (IP) with Flag antibodies and immunoblotted with the indicated antibodies.

Using a yeast two-hybrid assay to screen a mouse brain cDNA library, we identified Dorfin/Rnf19a as a novel binding partner of PSD-95. Dorfin contains two RING domains separated by the IBR domain in the N-terminal half followed by two putative transmembrane domains and a C-terminal PDZ domain-binding motif (Fig. 1A). The last seven amino acids of Dorfin interacted with the PDZ1 and PDZ2, but not PDZ3, domains of PSD-95 (Fig. 1B). Dorfin also interacted with PDZ domains from PSD-95 relatives (PSD-93/Chapsyn-110, SAP97, SAP102), but not with those from S-SCAM, GRIP2, or Shank1. A mutant Dorfin, in which the last amino acid residue was mutated (I840A), failed to interact with PSD-95, indicative of a canonical PDZ interaction. These PDZ interactions were verified by GST-pull down assays, which showed that full-length PSD-95 family proteins expressed in heterologous cells were pulled down by GST-Dorfin, although PSD-93 and SAP102 showed weaker interactions relative to PSD-95 and SAP97 (Fig. 1C). *In vitro* coimmunoprecipitation experiments independently confirmed the PDZ-dependent interactions of Dorfin with PSD-95 and SAP97 (Fig. 1D,E).

### Generation of *Dorfin* ^−/−^ mice

**Figure 2:**
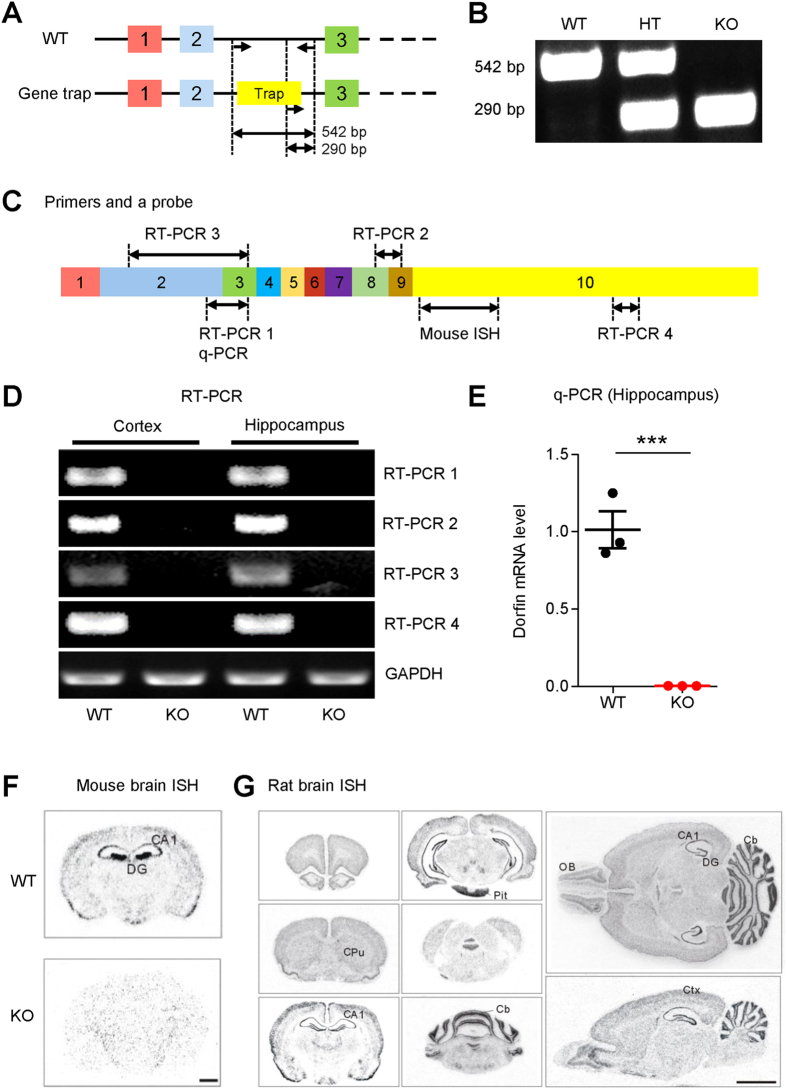
Generation of *Dorfin*^−/−^ mice. (**A**) Diagram depicting the structure of the *Dorfin* gene showing exons and introns, and the site of gene trap insertion in the intron between exon 2 and exon 3. (**B**) Identification of the trapped *Dorfin* gene by PCR genotyping. (**C**) Locations of the primers for RT-PCR and qPCR and a probe for *in situ* hybridization in the mouse *Dorfin* gene. (**D**,**E**) Undetectable expression of *Dorfin* mRNA in the *Dorfin*^−/−^ brain (2 months) by conventional RT-PCR and qPCR. (**F**) Distribution patterns of Dorfin mRNAs in WT and *Dorfin*^−/−^ mouse brain sections (2 months) revealed by *in situ* hybridization. CA1 and DG, CA1 and DG regions of the hippocampus. Scale bar, 1 mm. (**G**) Distribution patterns of *Dorfin* mRNA in WT rat brain sections (3 months) revealed by *in situ* hybridization. Cb, cerebellum; CPu, caudate-putamen; Ctx, cerebral cortex; OB, olfactory bulb; Pit, pituitary gland. Scale bar, 5 mm.

In order to explore the *in vivo* functions of Dorfin, we generated *Dorfin*^−/−^ mice using a gene-trap approach in which the intron between exons 2 and 3 of the *Dorfin* gene was targeted (Fig. 2A). The trapped allele was verified by genotyping polymerase chain reaction (PCR) (Fig. 2B). Reverse transcription PCR (RT-PCR) and quantitative PCR (qPCR) revealed that Dorfin mRNA was undetectable in both the cortex and hippocampus of *Dorfin*^−/−^ mice (Fig. 2C–E).

*In situ* hybridization experiments on wild-type (WT) and *Dorfin*^−/−^ brains indicated that Dorfin mRNA is widely expressed in various brain regions of WT mice, including the cortex and hippocampus, whereas signals were almost undetectable in the *Dorfin*^−/−^ brain (Fig. 2F). Widespread Dorfin mRNA signals were also observed in rat brains (Fig. 2G). Notably, Dorfin mRNA signals in the hippocampus were stronger in the DG region than in the CA1 region, especially in the mouse brain. Our results are largely in agreement with data from the Allen Brain Atlas (http://www.brain-map.org/) for mouse Dorfin mRNA. Despite repeated attempts, we could not characterize expression/distribution patterns of Dorfin protein because suitable antibodies are lacking.

### General characterization of *Dorfin* ^−/−^ mice

**Figure 3:**
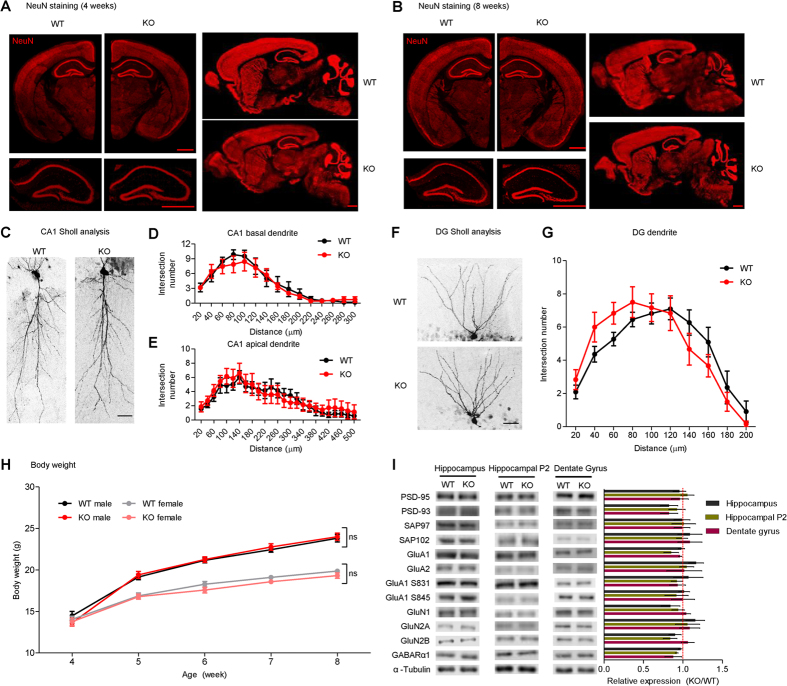
General characterization of *Dorfin*^−/−^ mice. (**A**,**B**) Normal gross morphology of the *Dorfin*^−/−^ brain at postnatal weeks 4 and 8, as determined by staining for the neuronal marker NeuN in coronal and sagittal sections. Scale bar, 1.0 mm. (**C–G**) Normal dendritic morphology of principal neurons in hippocampal CA1 and DG regions (2 weeks), as demonstrated by biocytin injection and Sholl analysis. Scale bar, 50 μm. (s.e.m., CA1 basal, n = 6 cells from 4 mice for WT and 7, 5 for KO; CA1 apical, n = 7, 4 for WT and 7, 5 for KO; DG, n = 7, 5 for WT and 6, 5 for KO). (**H**) Normal body weights of male and female *Dorfin*^−/−^ mice during postnatal weeks 4***–***8. (s.e.m., n = 17 for WT male, 19 for KO male, 17 for WT female, and 14 for KO female). (**I**) Normal levels of synaptic scaffolding and receptor proteins in the *Dorfin*^−/−^ hippocampus (2–3 months), as demonstrated by immunoblot analysis of whole hippocampal lysates, the crude synaptosomal (P2) hippocampal fraction, and microdissected whole DG lysates (s.e.m., n = 3***–***6 for WT and KO).

Immunohistochemical staining of WT and *Dorfin*^−/−^ brains using antibodies to NeuN, a neuronal marker, revealed that *Dorfin*^−/−^ brains have normal gross morphology at postnatal weeks 4 and 8 (Fig. 3A,B). In addition, a Sholl analysis of biocytin-injected neurons indicated that *Dorfin*^−/−^ principal neurons in the DG and CA1 regions of the hippocampus have normal dendritic morphology (Fig. 3C–G). *Dorfin*^−/−^ mice grew normally during postnatal weeks 4–8, with both males and females displaying body weights comparable to those of WT mice (Fig. 3H). In addition, immunoblot analyses of hippocampal lysates, the hippocampal crude synaptosomal fraction, and microdissected DG lysates revealed that levels of synaptic scaffolding and receptor proteins were not different between the genotypes (Fig. 3I).

### *Dorfin* ^−/−^ mice show increased mEPSC amplitude and enhanced LTP in the hippocampal DG region

**Figure 4:**
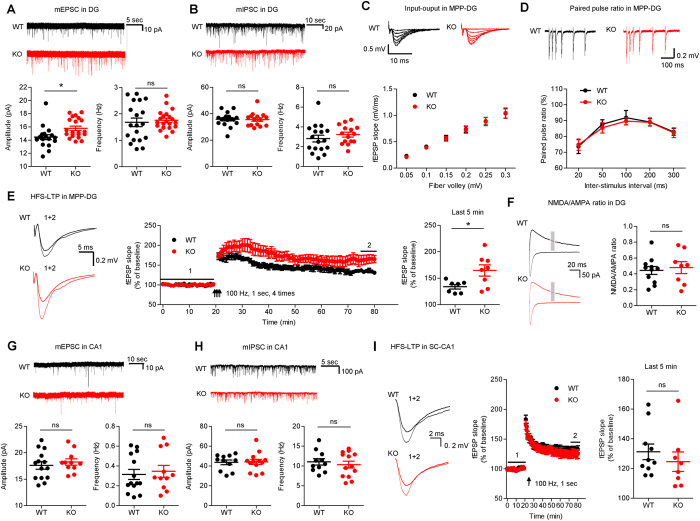
Enhanced LTP in the DG, but not CA1, region of the *Dorfin*^−/−^ hippocampus. (**A**) Increased amplitude, but normal frequency, of mEPSCs in *Dorfin*^−/−^ DG granule cells (P15–17, s.e.m., n = 19 cells from 5 mice for WT and 21, 3 for KO, *p < 0.05, ns, not significant, Student’s t-test). (**B**) Normal frequency and amplitude of mIPSCs in *Dorfin*^−/−^ DG granule cells (P15–17, s.e.m., n = 17, 4 for WT and 15, 4 for KO, ns, not significant, Student’s t-test). (**C**) Normal input-output curve, determined by plotting field EPSP (fEPSP) slopes against fiber volley amplitudes at *Dorfin*^−/−^ MPP-DG synapses (3 weeks, n = 11 slices from 4 mice for WT and 13, 4 for KO). (**D**) Normal paired pulse ratio, determined by plotting the ratios of two consecutive fEPSP slopes against inter-stimulus intervals at *Dorfin*^−/−^ MPP-DG synapses (3 weeks, n = 11 slices from 4 WT mice and 13, 4 for KO). (**E**) Enhanced LTP induced by four trains of HFS at *Dorfin*^−/−^ MPP-DG synapses (4 weeks, s.e.m., n = 7 slices from 4 mice for WT and 8, 7 for KO, *p < 0.05, Student’s t-test). (**F**) Normal NMDA/AMPA ratio at *Dorfin*^−/−^ MPP-DG synapses, determined by comparing evoked fEPSP slopes at the holding potentials of −70 (AMPA) and +40 (NMDA) mV (3 weeks, s.e.m., n = 11 cells from 6 mice for WT and 8, 4 for KO, ns, not significant, Student’s t-test). (**G**) Normal frequency and amplitude of mEPSCs in *Dorfin*^−/−^ CA1 pyramidal cells (P15–17, s.e.m., n = 14 cells from 3 mice for WT and 11, 3 for KO, ns, not significant, Student’s t-test). (**H**) Normal frequency and amplitude of mIPSCs in *Dorfin*^−/−^ CA1 pyramidal cells (P15–20, s.e.m., n = 11, 3 for WT and 13, 4 for KO, ns, not significant, Student’s t-test). (**J**) Normal LTP induced by HFS at *Dorfin*^−/−^ SC-CA1 synapses (4 weeks, n = 10 slices from 6 mice for WT and 8, 5 for KO, ns, not significant, Student’s t-test).

Because Dorfin interacts with PSD-95, an abundant excitatory synaptic scaffolding protein, we explored possible alterations in excitatory synaptic transmission in *Dorfin*^−/−^ mice. Granule cells in the *Dorfin*^−/−^ DG region showed an increase in the amplitude, but not frequency, of miniature excitatory postsynaptic currents (mEPSCs) compared with WT neurons (Fig. 4A). In contrast, both the frequency and amplitude of miniature inhibitory postsynaptic currents (mIPSCs) were normal in these neurons (Fig. 4B). In addition, the input–output curve at *Dorfin*^−/−^ medial perforant pathway synapses on DG granule cells (MPP-DG synapses) was normal, as measured by the slopes of field EPSPs (fEPSPs) plotted against fiber volley amplitudes (Fig. 4C). Lastly, paired-pulse ratio plots against inter-stimulus intervals were normal (Fig. 4D), suggestive of unaltered presynaptic release probability.

Intriguingly, LTP at MPP-DG synapses induced by four trains of high-frequency stimulation (HFS; 100 Hz) was significantly enhanced in *Dorfin*^−/−^ mice compared with WT mice (Fig. 4E). This does not seem to be attributable to an increase in NMDAR function because the ratio of NMDAR- and AMPAR-mediated currents at these synapses was not changed (Fig. 4F), although LTP at MPP-DG synapses is known to be dependent on NMDARs^41^,^42^.

Because the CA1 region of the hippocampus also expresses Dorfin mRNA, although to a lesser extent than the DG, we tested possible changes in mE/IPSCs and LTP in this region. We found that neither mEPSCs nor mIPSCs were altered in *Dorfin*^−/−^ CA1 pyramidal neurons (Fig. 4G,H). In addition, LTP induced by HFS (100 Hz) was unaltered at *Dorfin*^−/−^ Schaffer collateral-CA1 pyramidal (SC-CA1) synapses (Fig. 4I). These results suggest that Dorfin deletion leads to an enhancement in LTP at MPP-DG, but not SC-CA1, synapses.

### *Dorfin* ^−/−^ mice are hyperactive in a familiar, but not in a novel, environment

**Figure 5:**
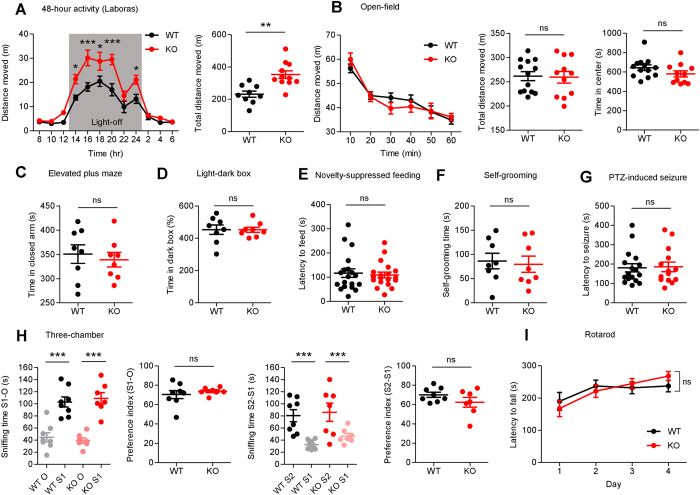
*Dorfin*^−/−^ mice display hyperactivity in a familiar, but not in a novel, environment, and show normal levels of anxiety-like behavior, self-grooming, seizure propensity, social interaction, and motor coordination. (**A**) *Dorfin*^−/−^ mice display enhanced locomotor activity in a familiar environment, as measured by 48-hour continuous monitoring of mouse movements in a familiar environment averaged in a 24-hour cycle (Laboras, n = 9 mice for WT and 11 for KO, two-way ANOVA, Bonferroni post-hoc, ***p < 0.001, **p < 0.01, *p < 0.05, Student’s t-test for total moved distance). (**B**) *Dorfin*^−/−^ mice show normal locomotor activity in the open field test (novel environment). Note that the time spent in the center region is also normal. (s.e.m., n = 13 mice for WT and 11 for KO, ns, not significant, two-way ANOVA, Bonferroni post-hoc, Student’s t-test). (**C–E**) *Dorfin*^−/−^ mice display normal levels of anxiety-like behavior in elevated plus maze (**C**), light-dark box (**D**), and novelty-suppressed feeding tests (**E**) (s.e.m, n = 8 mice for WT and KO in elevated plus-maze and light-dark box tests and 19 for WT and KO mice in the novelty-suppressed feeding test, ns, not significant, Student’s t-test). (**F–I**) *Dorfin*^−/−^ mice show normal levels of self-grooming (**F**), PTZ-induced seizure (**G**), three-chamber social interaction (**H**), and rotarod motor coordination **(I)** (s.e.m., n = 8 mice for WT and KO [self-grooming], 17 for WT and 14 for KO [seizure], 8 for WT and KO [three-chamber], and 12 for WT and KO [rotarod], ns, not significant, Student’s t-test).

We next explored whether *Dorfin*^−/−^ mice show any behavioral abnormalities. *Dorfin*^−/−^ mice exhibited enhanced locomotor activity during the light-off phase in a familiar environment, as measured by 48-hour continuous monitoring of movements in a familiar environment in which mice had been habituated for 3 days before the test (Fig. 5A). In contrast, *Dorfin*^−/−^ mice showed normal locomotion in a novel environment, measured by the open field test (Fig. 5B), suggesting that *Dorfin*^−/−^ mice are hyperactive in a familiar, but not a novel, environment.

We next tested whether *Dorfin*^−/−^ mice display anxiety-like behaviors by three different tests. *Dorfin*^−/−^ mice spent a normal amount of time in the center region of the open field arena (Fig. 5B), in the open/closed arm of the elevated plus-maze (Fig. 5C), and in the light-dark chamber of the light-dark box (Fig. 5D). In the novelty-suppressed feeding test, WT and *Dorfin*^−/−^ mice showed comparable levels of latency to feed in a novel, open field (Fig. 5E). These results indicate that *Dorfin*^−/−^ mice do not display anxiety-like behaviors.

Lastly, *Dorfin*^−/−^ mice showed normal levels of self-grooming behavior (Fig. 5F), pentylenetetrazole (PTZ)-induced seizure (Fig. 5G), social interaction and social novelty recognition in the three-chamber test (Fig. 5H), and motor coordination in the rotarod test (Fig. 5I). These results collectively indicate that *Dorfin*^−/−^ mice show a specific increase in locomotor activity in a familiar, but not a novel, environment, whereas other tested behaviors are normal.

### *Dorfin* ^−/−^ mice show a specific deficit in contextual fear conditioning, but not in other types of learning and memory

**Figure 6:**
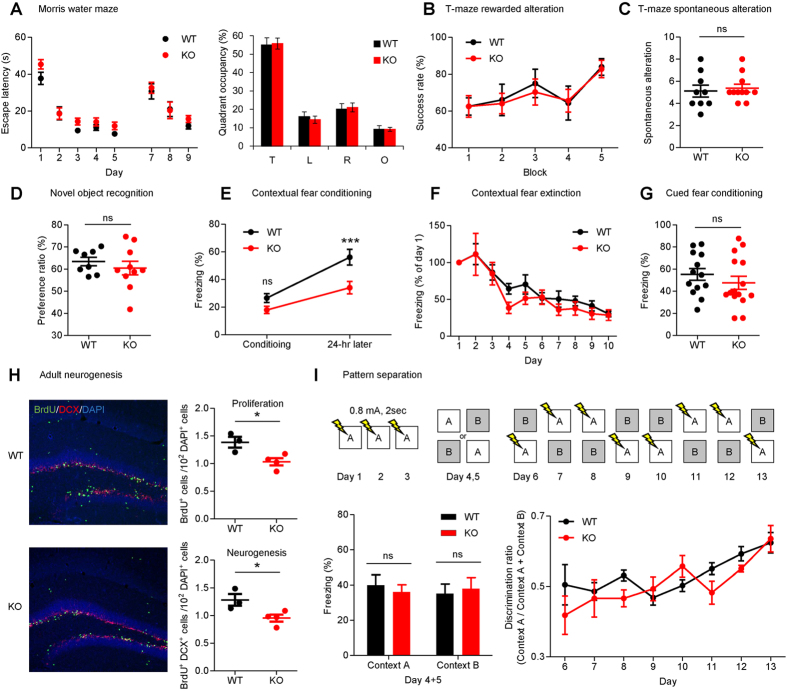
*Dorfin*^−/−^ mice display a specific deficit in contextual fear memory but not in other types of learning and memory behaviors. (**A**) *Dorfin*^−/−^ mice show normal spatial learning and memory in the learning, probe, and reversal phases of the Morris water maze test (T: target, L: left, R: right, O: opposite, s.e.m., n = 14 mice for WT and 16 for KO, ns, not significant, two-way ANOVA, Bonferroni post-hoc, Student’s t-test). (**B**,**C**) *Dorfin*^−/−^ mice display normal spatial working memory in the T-maze rewarded (**B**) and spontaneous (**C**) alteration tests (8 trials over 2 days constitutes one block; s.e.m., rewarded, n = 7 for WT and 8 for KO; spontaneous, n = 9 for WT and 11 for KO, ns, not significant, two-way ANOVA, Bonferroni post-hoc, Student’s t-test). (**D**) *Dorfin*^−/−^ mice display novel object preference comparable to that of WT mice (s.e.m, n = 8 for WT and 10 for KO, ns, not significant, Student’s t-test). (**E–G**) *Dorfin*^−/−^ mice display reduced contextual fear memory but normal fear extinction and cued fear conditioning. (s.e.m., contextual fear conditioning, n = 16 for WT and KO; contextual fear extinction, n = 16 for WT and KO; cued fear conditioning, n = 13 for WT and 15 for KO, ns, not significant, ***p < 0.001, two-way ANOVA, Bonferroni post-hoc, Student’s t-test). (**H**) *Dorfin*^−/−^ mice show suppressed neurogenesis in the adult DG. Hippocampal slices from BrdU-injected mice (6 weeks) were triply labeled for BrdU, doublecortin (DCX), and DAPI, and the numbers of BrdU-positive cells as well as cells doubly positive for BrdU and DCX normalized to DAPI-positive cells were quantified. Scale bar, 50 μm. (s.e.m., n = 3 mice for WT and n = 4 for KO, *p < 0.05, Student’s t-test). (**I**) Normal pattern separation in *Dorfin*^−/−^ mice. (s.e.m., n = 9 mice for WT and n = 7 for KO, ns, not significant, Student’s t-test, two-way ANOVA, Bonferroni post-hoc).

Because *Dorfin*^−/−^ mice show enhanced LTP in the hippocampal DG region, we subjected *Dorfin*^−/−^ mice to a battery of learning and memory behavioral tests. In the Morris water maze test, which measures spatial learning and memory^43^, *Dorfin*^−/−^ mice performed normally during the learning, probe, and reversal phases (Fig. 6A). *Dorfin*^−/−^ mice also performed normally in the T-maze rewarded alteration and T-maze spontaneous alteration tests, which measure spatial working memory^44^ (Fig. 6B,C). In the novel object recognition test, which measures object recognition memory and novel object preference, WT and *Dorfin*^−/−^ mice showed similar preference for the novel object (Fig. 6D).

In the contextual fear-conditioning test, *Dorfin*^−/−^ mice showed reduced freezing in the same shock chamber 24 hours after conditioning compared with WT mice (Fig. 6E). In contrast, *Dorfin*^−/−^ mice showed contextual fear extinction and cued fear conditioning comparable to those of WT mice (Fig. 6F,G). These results collectively suggest that *Dorfin*^−/−^ mice have a specific deficit in contextual fear conditioning, but not in other types of learning and memory.

### *Dorfin* ^−/−^ mice display suppressed neurogenesis in the adult DG but normal pattern separation

The increases in mEPSC amplitude and LTP observed in the DG region of *Dorfin*^−/−^ mice might be associated with alterations in the adult neurogenesis, known to regulate synaptic plasticity in the DG^45^. When cells in the inner border region of the DG granule cell layer were labeled with BrdU, a marker of dividing cells, the number of BrdU-positive cells was significantly reduced in the adult *Dorfin*^−/−^ DG (6 weeks), compared with that in WT mice (Fig. 6H). In addition, the number of cells doubly positive for BrdU and doublecortin, a marker of newly generated neurons, was significantly reduced in the *Dorfin*^−/−^ DG. These results indicate that Dorfin deletion leads to the suppression of neurogenesis in the adult DG.

Neurogenesis in the adult DG has been associated with spatial pattern separation in addition to spatial/contextual learning and memory^45^,^46^,^47^. We thus subjected *Dorfin*^−/−^ mice to the contextual discrimination test and found that *Dorfin*^−/−^ mice could normally learn to discriminate two similar contexts, in which one is associated with foot shock (Fig. 6I).

### Proteomic identification of under-ubiquitinated proteins in the *Dorfin* ^−/−^ brain

**Figure 7:**
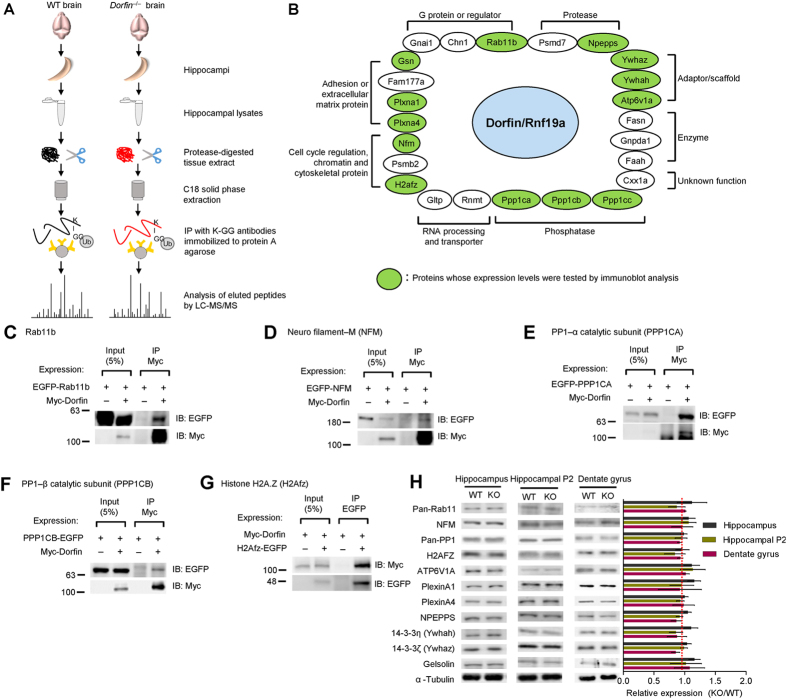
Proteomic identification of proteins with reduced ubiquitination in the *Dorfin*^−/−^ hippocampus. (**A**) Procedures for the identification of proteins with altered ubiquitination in the *Dorfin*^−/−^ brain. Ubiquitinated peptides from hippocampus lysates were immuno-affinity purified with the K-GG antibody, followed by tandem mass spectrometry (LC-MS/MS) analysis for qualitative sequencing, target site identification, and quantification. (n = 6 mice for WT and 8 for KO in one sample respectively). (**B**) List of proteins whose ubiquitination levels are decreased by >2.5 folds in the *Dorfin*^−/−^ hippocampus relative to WT controls (see also Supplementary Table 1). Green circles indicate the proteins whose expression levels were tested by immunoblot of hippocampal lysates (see also Fig. 7H). (**C**–**G**) Dorfin forms a complex with five selected proteins from the list in Fig. 7B. HEK293 cells doubly expressing Myc-Dorfin and the indicated proteins were immunoprecipitated and immunoblotted with Myc or EGFP antibodies. Rab11b (**C**), NFM (**D**), PPP1CA (**E**), PPP1CB (**F**) and H2AFZ (**G**). Input, 5%. (**H**) Protein expression levels in the *Dorfin*^−/−^ hippocampus (2–3 months) relative to those in WT controls, as determined by the immunoblot analysis of whole hippocampal lysates, the crude synaptosomal (P2) hippocampal fraction, and microdissected whole DG lysates. (s.e.m., n = 3***–***6 for WT and KO hippocampi).

If Dorfin acts as a ubiquitin ligase in the brain, we should be able to observe decreases in the ubiquitination levels of Dorfin substrate proteins in the *Dorfin*^−/−^ brain. To this end, we immunoprecipitated hippocampal lysates using an antibody that targets the K-GG peptide in ubiquitinated proteins, followed by liquid chromatography-tandem mass spectrometric (LC-MS/MS) analyses of the precipitates (Fig. 7A).

We sorted positive proteins in order of greater decreases in ubiquitination, generating a list of 24 proteins whose ubiquitination levels were substantially decreased (>2.5 fold) in the *Dorfin*^−/−^ brain relative to WT controls (Supplemental Table S1). These proteins could be classified into several functional groups, including adhesion molecules/extracellular matrix, adaptor/scaffolds, G protein-related proteins, phosphatases, proteases, enzymes, and cell cycle/chromatin regulatory proteins (Fig. 7B).

Using coimmunoprecipitation experiments in heterologous cells, we verified the association of some of these proteins with Dorfin (Fig. 7C–G). These proteins included Rab11b (a small GTPase), neurofilament M, the α and β subunits of PP1 phosphatase, and histone H2A. Unexpectedly, none of these five proteins were found to be ubiquitinated when coexpressed with Dorfin in heterologous cells (Supplementary Fig. 1). In addition, their protein levels were unaltered in *Dorfin*^−/−^ mice, as measured by immunoblot analysis of hippocampal lysates, the hippocampal crude synaptosomal fraction, and microdissected DG lysates (Fig. 7H). In addition, there were no changes in the expression levels of other proteins in the list, including ATP6V1A, Plexin A1/A4, NPEPPS, 14-3-3 η/ζ, and gelsolin (Fig. 7H). Therefore, Dorfin deletion *in vivo* does not seem to cause detectable decreases in the levels of the tested proteins.

## Discussion

Our study indicates that Dorfin deletion in mice leads to decreased neurogenesis in the DG, enhanced LTP in the MPP-DG pathway, and a specific behavioral deficit in contextual fear conditioning, but not in other types of learning and memory. We also identified Dorfin as a novel binding partner of PSD-95, and identified a number of proteins whose ubiquitination levels are decreased in *Dorfin*^−/−^ mice.

The functional significance of the interaction between Dorfin and PSD-95 was not directly explored in the present study. However, given that PSD-95 is an abundant scaffolding protein enriched at excitatory synapses, it is possible that PSD-95 may promote synaptic targeting of Dorfin, as demonstrated for many other PSD-95-interacting proteins^48^,^49^. Alternatively, because PSD-95 is a multi-domain protein that interacts with diverse membrane, signaling, and scaffolding/adaptor proteins^49^, and E3 ligases often interact with adaptor/scaffolding proteins to broaden the spectrum of substrate proteins^1^,^2^,^3^,^4^,^5^, PSD-95 might function as a gateway through which Dorfin interacts with diverse substrates. This is reminiscent of the interaction between the synaptic PDZ protein CASK/LIN-2 and Parkin^50^, an E3 ubiquitin ligase known to negatively regulate excitatory synapse number and strength^51^ and protect postmitotic neurons from excitotoxicity^52^. Another example is the interaction of Piccolo and Bassoon, two large active zone proteins controlling presynaptic protein assembly^53^, with Siah1 (an E3 ubiquitin ligase), which is thought to promote the maintenance of synaptic integrity by modulating presynaptic protein degradation^30^.

*Dorfin*^−/−^ mice displayed two synaptic phenotypes in the hippocampus: increased mEPSC amplitude in DG granule cells and enhanced HFS-induced LTP at MPP-DG synapses. These results suggest that Dorfin may have negative influences on the synaptic AMPARs as well as on LTP. Behaviorally, *Dorfin*^−/−^ mice showed a specific impairment in contextual fear conditioning, but not in other types of learning and memory, including spatial learning and memory (Morris water maze), novel object recognition memory, spatial working memory (T maze), fear extinction, cued fear conditioning, and pattern separation.

It is unclear how Dorfin deletion leads to increases in mEPSC amplitude and LTP in the DG, although they likely involve increases in the number or function of AMPARs under basal conditions or during LTP. Phosphorylation of two sites on the GluA1 subunit of AMPARs, S831 and S845, have been implicated in the regulation of the biophysical properties of AMPARs and AMPAR-mediated synaptic plasticity^54^,^55^. However, our data indicated that the levels of these phosphorylations were unaltered in the *Dorfin*^−/−^ brain, excluding this possibility.

Our data provide additional support for the notion that E3 ubiquitin ligases regulate synaptic plasticity and learning and memory. It has been shown that a maternal deficiency of Ube3A (ubiquitin protein ligase E3A) suppresses LTP and impairs contextual fear conditioning in mice^56^. In addition, Praja2 (praja ring finger 2, E3 ubiquitin protein ligase) knockdown in rats suppresses late LTP^57^. Late LTP and contextual fear conditioning are suppressed in *Mindbomb-1*^−/−^ mice^58^ and *APC*/*C-Cdh1*^−/−^ mice^59^,^60^. Lastly, enhanced LTP and suppressed fear conditioning are observed in *Scrapper*^−/−^ and *Scrapper*^+/−^ mice, respectively^61^,^62^.

Our results are unique in that an E3 deletion in the DG (not CA1) region of the hippocampus alters LTP and learning and memory. This is in line with the reported association of hippocampal DG granule cells with spatial behavior and contextual fear conditioning^63^,^64^,^65^. In addition, this suggests that E3 ligases in diverse brain regions in addition to the hippocampal CA1 region are important for the regulation of synaptic plasticity, as previously observed in the amygdala of *APC*/*C-Cdh1*^−/−^ mice^66^, striatum of *Parkin*^−/−^ mice^67^, and visual cortex of *Ube3a*^*m−*/*p+*^ mice^68^.

A notable finding was that enhanced—not reduced—LTP in *Dorfin*^−/−^ mice was associated with impaired contextual fear conditioning, contrary to our expectations. However, previous studies on mice lacking, for example, Fmr2, Gα1, PDE4D, IRSp53, and SCRAPPER proteins, have linked enhanced hippocampal LTP in the Schaffer collateral pathway with decreased contextual fear conditioning^61^,^62^,^69^,^70^,^71^,^72^,^73^. These results, together with ours, suggest that deviation of hippocampal LTP in either direction in the DG or CA1 region may cause similar impairments in learning and memory.

Although the mechanisms leading to enhanced LTP and impaired contextual fear conditioning in *Dorfin*^−/−^ mice remain to be determined, the list of proteins whose ubiquitination levels were reduced by >2.5-fold in *Dorfin*^−/−^ mice might provide some starting points. Indeed, many of these proteins are enriched in the postsynaptic density (PSD) and have been implicated in the regulation of excitatory synaptic transmission, synaptic plasticity, and learning and memory (Supplemental Table S1). For example, Rab11a/b promotes translocation of recycling endosomes into dendritic spines and local exocytosis of GluA1 during LTP^74^,^75^,^76^,^77^. Therefore, Dorfin-dependent ubiquitination and degradation of Rab11a/b proteins would negatively regulate LTP. In addition, transgenic mice in which the signaling adaptors 14-3-3η and 14-3-3ζ are functionally inhibited by genetic expression of an inhibitor protein display reductions in synaptic content of NMDARs, NMDAR-mediated synaptic transmission, LTP, and contextual fear conditioning^78^, suggesting that 14-3-3η and 14-3-3ζ positively regulate synaptic plasticity and learning and memory. Therefore, these functions may be suppressed by Dorfin-dependent 14-3-3 protein degradation.

Contrary to our initial expectation, our data indicate that some of the proteins that exhibited decreased ubiquitination in *Dorfin*^−/−^ mice were expressed at normal levels in the mutant brain, despite our demonstration that they associate with Dorfin *in vitro*. However, protein ubiquitination has been shown to have functions other than protein degradation, such as modulation of protein function, endocytosis and subcellular localization, as well as protein-protein interactions^1^,^2^,^3^,^4^,^5^. Our data indicate that at least the five proteins that we demonstrated to form a complex with Dorfin (Rab11b, neurofilament-M, PP1-α catalytic subunit, PP1-β catalytic subunit, and histone H2A.Z) are not directly ubiquitinated in heterologous cells. Given that these proteins were found to be less ubiquitinated in the *Dorfin*^−/−^ hippocampus through an unbiased screen, their ubiquitination might require certain conditions unique to neuronal/brain environments, or some proteins other than these five proteins can be ubiquitinated by Dorfin in heterologous cells.

Lastly, our results indicate that neurogenesis in the *Dorfin*^−/−^ adult DG is suppressed. Because adult neurogenesis in the DG has been implicated in the regulation of synaptic plasticity at DG synapses and hippocampus-dependent behaviors including pattern separation and spatial/contextual learning and memory^45^,^46^,^47^,^79^, the reduced neurogenesis in the *Dorfin*^−/−^ adult DG might be associated with the enhanced LTP and impaired contextual fear conditioning in these mice. However, because reduced adult neurogenesis has been shown to suppress both LTP and LTD^80^, our results, where reduced neurogenesis was associated with enhanced (but not suppressed) LTP, differ from the previous results. However, reduced neurogenesis in the adult DG has been associated with reduced performance in contextual fear conditioning in previous studies^45^,^81^, being in line with our results.

In conclusion, our study identifies the E3 ubiquitin ligase Dorfin as a novel ligand of PSD-95, and provides *in vivo* evidence supporting the hypothesis that Dorfin may regulate adult neurogenesis, excitatory synaptic transmission, synaptic plasticity, and contextual fear conditioning.

## Methods

### DNA constructs

Full-length Dorfin cDNA was amplified by PCR from a rat brain cDNA library and subcloned into GW1 (British Biotechnology). For Flag-Dorfin, rat Dorfin in GW1 was PCR amplified and subcloned into p3XFLAG-CMV-7.1 vector (Sigma). For Myc-Dorfin, full-length rat Dorfin was subcloned into pGW1-Myc. Full-length PSD-95, EGFP-PSD-93, EGFP-SAP97, EGFP-SAP102, EGFP-S-SCAM, Myc-GRIP2 for GST pull down have been described^82^. Full-length pCFP-Rab11b was a kind gift from Dr. Wondo Heo at KAIST. Full-length pEGFP-mNFM, pEGFP-PP1α, pEGFP-PP1β, and pRK5-HA-Ub were obtained from Addgene (ID # 32909, 44224, 44223, and 17608 respectively). pEGFP-H2Afz was amplified by PCR from a mouse brain cDNA library and subcloned into pEGFP-N1.

### Antibodies

The following antibodies have been described: EGFP^83^, PSD-93, SAP97, SAP102, S-SCAM^84^, and GluA1^85^. The following antibodies were purchased from commercial sources: mouse monoclonal PSD-95 K28/43, GluN2B N59/36 (UC Davis/NIH NeuroMab Facility) NR1/GluN1 (BD Pharmingen), GluN2A, NPEPPS, NeuN (Millipore), pGluR1 S831 and S845 (Cell Signaling), GluA2, rabbit polyclonal and mouse monoclonal Myc, ATP6V1A, Rab11, 14-3-3η, 14-3-3ζ, PP1, ubiquitin (Santa Cruz Biotechnology), mouse monoclonal HA (Roche Molecular Biochemicals), mouse monoclonal Flag M2 (Sigma), α-tubulin (Sigma), GABARα1 (SySy), doublecortin, plexin A1 and A4, gelsolin (Abcam), BrdU (Novus Biologicals). Immuno-blot assays were performed using Odyssey Fc (LI-COR Biosciences).

### Yeast two-hybrid constructs and assays

The last 7 amino acids of mouse Dorfin (aa 834–840, WT and I840A) were generated by annealing oligonucleotides and subcloning them into pBHA. The PDZ domains of various proteins were subcloned into pGAD10 (a prey vector: Clontech) as described previously^86^. Yeast two-hybrid assays were performed as previously described^87^.

### GST pull down assay

For pull down, the last seven amino acids of mouse Dorfin (aa 834–840, WT and I840A) were generated by annealing oligonucleotides and subcloning them into pGEX-4T-1 (Amersham Pharmacia Biotech). For pull down, HEK293T cells were transfected with PSD-95, EGFP-SAP97, EGFP-PSD-93, EGFP-SAP102, EGFP-S-SCAM, Myc-GRIP2. Pulled down precipitates were analyzed by immunoblotting with PSD-95, EGFP, and Myc antibodies.

### *In situ* hybridization

*In situ* hybridization was performed as previously described^82^. Hybridization probes for mouse Dorfin mRNA were prepared using nt 2161–2820 of mouse Dorfin (NM_013923.2) and subcloned into pGEM-7Zf vector. Hybridization probes for rat Dorfin mRNA were prepared using nt 2609–3187 of rat Dorfin (NM_001130560.1) and subcloned into pGEM-7Zf vector.

### Ubiquitination assay

Myc-Dorfin and HA-ubiquitin were triply, or doubly (without Dorfin), transfected with EGFP-Rab11b, EGFP-NFM, EGFP-PPP1CA, PPP1CB-EGFP, or H2Afz-EGFP into HEK293 cells. Cell lysates (2% SDS, 150 mM NaCl, 10 mM Tris-HCl) diluted to a dilution buffer (10 mM Tris-HCl, 150 mM NaCl, 2 mM EDTA, 1% Triton X-100) were incubated with EGFP antibodies, followed by immunoprecipitation and immunoblot analyses.

### Animals

*Dorfin*^−/−^ mice were generated from mouse sperm with the gene trap cassette inserted into the intron between exon 2 and exon 3 of the *Dorfin* gene, obtained from TIGM (OST244733). Mice in the genetic background of 129/SvEv were backcrossed with C57BL6 mice for more than five generations. Mice for experiments were obtained by mating male and female heterozygous mice. Mice were maintained according to the Requirements of Animal Research at KAIST, and all procedures were approved by the Committee of Animal Research at KAIST (KA2012-19). All experiments involving mice were performed in accordance with relevant guidelines and regulations. Mice were housed in a standard cage environment under 12-hour light and dark cycles.

### Primers for genotyping PCR, RT-PCR and qPCR

Primers for genotyping PCR were 5′-CGG-GCT-GGG-TTT-ACA-TAG-AA-3′ (WT 5′), 5′-GAC-CCA-AAT-GTC-CAT-CAA-CC-3′ (WT 3′) and 5′-CAA-AAT-GGC-GTT-ACT-TAA-GC-3′ (Trap 5′). Primers for RT-PCR were consisted of 4 sets. Dorfin 1: 5′-ACT-GAA-CGG-TTT-AAT-CCT-C-3′ and 5′-CTG-TTC-CAC-AGC-CTT-CTC-3′. Dorfin 2: 5′-TGT-TTC-AAC-AGG-TTG-GAA-GTA-3′ and 5′-GCG-TTA-TCA-CTA-ACT-GTT-CCC-AAG-3′. Dorfin 3: 5′-ATC-AAG-GGA-GCT-CAA-TGG-TGG-3′ and 5′-GCA-TCA-CAG-GTC-TGG-TTT-GG-3′Dorfin 4: 5′-GCA-AGA-TGG-TGG-CAG-TTT-TT-3′ and 5′-CTA-ATG-GGA-CCG-TCT-TCT-GC-3′. Primers for qPCR were 5′-ACT-GAA-CGG-TTT-AAT-CCT-C-3′ and 5′-CTG-TTC-CAC-AGC-CTT-CTC-3′.

### Immunohistochemistry

Brain slices were prepared from mice at 4 and 8 weeks. Brain sections (50 μm) were permeablized with 0.5% TritonX-100 for 30 min and stained with the NeuN antibody. After 24 hours, sections were stained with secondary antibodies for 2 hours, followed by image acquisition with a confocal microscope (LSM780, Carl Zeiss).

### BrdU labeling

5-Bromo-2′-deoxyuridine (BrdU, Sigma) was injected intraperitoneally (50 mg/kg) to mice (6 weeks) for 3 days. Five days after the last injection, mice were perfused with 4% paraformaldehyde. Fixed brain slices (50 μm) obtained by vibratome were incubated in 1N HCl for 10 min on ice, followed by 2N HCl for 10 min at RT before moving them to 37 °C incubator for 20 min. Borate buffer (0.1 M) was added to the slices for 12 min at RT. Slices were then incubated with BrdU and DCX antibodies at 4 °C for 12 hours, followed by secondary antibody staining and mounting using Vectashield and DAPI. Slice images were acquired by confocal microscopy (LSM780, Zeiss).

### Sholl analysis

Pyramidal neurons in the CA1 region and granule cells in the DG region of the hippocampus were randomly chosen and infused with biocytin (0.5%) for 5 min. After injection, the slices were collected and fixed with 4% paraformaldehyde for 24 hours. The slices were stained by streptavidin antibody. Images were captured using 20× objectives (LSM 780, Carl Zeiss). Dendritic branch intersections were analyzed manually in a blind manner.

### Hippocampal/DG lysates and subcellular fractions

The crude synaptosomal (P2) fraction of adult mouse hippocampi (2–3 months) was prepared as described previously^88^. Whole hippocampal/DG lysates were prepared using homogenization buffer (0.32 M sucrose, 10 mM HEPES, 2 mM EDTA). For DG microdissection, we used vibratome to cut brain slices (400 μm) and dissect DG.

### Proteomic ubiquitination analysis

Hippocampal tissues were frozen in liquid nitrogen, followed by the Ubiscan analysis (Cell signaling) using K-GG peptide immunoprecipitation and LC-MS/MS (LTQ-Orbitrap-Velos and ESI-CID). MS/MS spectra were analyzed using SEQUEST 3G and the SORCERER 2 platform from Sage-N Research (v 4.0, Milpitas CA).

### Electrophysiology

Sagittal slices of hippocampus (300 μm for whole cell recordings, 400 μm for field recordings) were prepared using vibratome (VT1200S, Leica) in ice-cold sectioning buffer (212 mM sucrose, 25 mM NaHCO_3_, 5 mM KCl, 1.25 mM NaH_2_PO_4_, 10 mM D-glucose, 2 mM sodium pyruvate, 1.2 mM sodium ascorbate, 3.5 mM MgCl_2_, and 0.5 mM CaCl_2_ bubbled with 95% O_2_/5% CO_2_) and recovered at 32 °C in aCSF (125 mM NaCl, 25 mM NaHCO_3_, 2.5 mM KCl, 1.25 mM NaH_2_PO_4_, 10 mM D-glucose, 1.3 mM MgCl_2_, and 2.5 mM CaCl_2_). After 30 min, slices were maintained at room temperature. Whole-cell patch clamp recordings and field potential recordings were performed as previously described^72^. Recordings were performed using Multiclamp 700B amplifier and Axopatch 200B. Data were analyzed using Clampfit 10.2 (Molecular Devices).

Whole cell recordings for mEPSCs and the NMDA/AMPA ratio were made with recording pipettes (3–5 MΩ) filled with internal solution (117 mM CsMeSO4, 10 mM TEA-Cl, 8 mM NaCl, 10 mM HEPES, 5 mM QX-314-Cl, 4 mM Mg-ATP, 0.3 mM Na-GTP, and 10 mM EGTA). For mIPSC measurements, pipettes was were filled with a solution containing 115 mM CsCl, 10 mM TEA-Cl, 8 mM NaCl, 10 mM HEPES, 5 mM QX-314-Cl, 4 mM Mg-ATP, 0.3 mM Na-GTP, 10 mM EGTA. All internal solutions were adjusted to acquire pH 7.3 and 290–300 mOsm. Signals were filtered at 1.0 kHz for mEPSCs and mIPSCs and 2.0 kHz for NMDA/AMPA ratio experiments. For mEPSCs, picrotoxin (50 μM) and TTX (0.5 μM) were added to aCSF. For mIPSC recording, picrotoxin (50 μM), NBQX (10 μM), and AP5 (25 μM) were added. For NMDA/AMPA ratio experiments, picrotoxin (100 μM) was added. mEPSC and mIPSC recordings were performed at a holding potential of −70 mV for 2 min. For NMDA/AMPA ratio experiments, EPSCs were evoked with a glass pipette containing aCSF positioned in the MPP-DG pathway. AMPA EPSCs were recorded at −70 mV. After obtaining 25–30 stable traces, holding potential was changed to +40 mV to record NMDA EPSCs (20 traces). NMDA EPSCs at 50 ms after stimulation were used for the NMDA/AMPA ratio.

Field potential recordings were made with recording pipettes filled with aCSF. For input/output and paired pulse ratio recordings, stable fEPEPs were recorded for 10 min before main experiments began. For input/output recordings, stimuli were gradually increased to induce fiber volley amplitudes of 0.05–0.3 mV/ms, and 3 traces per stimulus were recorded. For paired pulse ratio recordings, inter-stimulus intervals were 25, 50, 100, 200, and 300 ms. Recordings were repeated 3 times. For high-frequency stimulation LTP, picrotoxin (100 μM) was added to aCSF. After obtaining stable baseline responses for 20 min, a single stimulus of 100 Hz (1 sec) was given to the SC-CA1 pathway, and four trains of 100- Hz stimuli (each 1 sec; 20 sec inter-train interval) were given to the MPP-DG pathway. The responses were recorded for an hour.

### 48-hour continuous monitoring of movements

For long-term continuous monitoring of mouse movements, we used the LABORAS^TM^ system (Metris), designed to detect vibrations originating from the movements of a mouse in the cage. Mice were single isolated and habituated in LABORAS cages in the recording room for 3 days. After habituation, the LABORAS cage with a mouse was placed on top of a vibration-sensitive recording platform for 72 hours. The data from the last 48 hours were analyzed, and averaged to 24-hour cycles.

### Open field test

The mice were placed in a 40 × 40 × 40 cm box and recorded of their horizontal locomotor activity under complete darkness (~0 lux) for an hour. The center zone line was 10 cm apart from the edge. Movements were analyzed using Ethovision XT 10 (Noldus).

### Elevated plus-maze

The maze, elevated to a height of 50 cm from the floor, consisted of two open arms, two closed arms, and a center zone. Mice placed on the center zone were recorded of their movements for 7 min and analyzed of the time spent in closed or open arms manually.

### Light-dark box test

The box had a dimension of 12 × 30 × 20 cm for the light chamber (~250~300 lux) and 14 × 13 × 20 cm for the dark chamber (~0~5 lux). A mouse was placed in the center of the light chamber, followed by movement monitoring for 10 min. Time spent in the light or dark chamber was analyzed manually.

### Novelty-suppressed feeding test

Mice were starved for 24 hours and placed in novel a 40 × 40 × 40 cm box. A food pellet was place in the center of the box, and the latency to approach and eat the food was measured.

### Self-grooming behavior

Mice were placed in an empty home cage and recorded of self-grooming behavior for 10 min. Self-grooming behavior was defined as stroking or scratching of face, head, or body areas with forelimbs, or licking body parts. Time of self-grooming was measured in a blind manner.

### PTZ-induced seizure

Mice were injected of 50 μg/weight (g) pentylenetetrazole (PTZ) into the intraperitoneal cavity and recorded of seizures for 10 min. Seizure was defined as convulsive movements including clonic and/or tonic-cloninc seizures while lying on the side and/or wild jumping.

### Three-chamber test

This test was performed to measure social interaction and social novelty recognition^89^. The apparatus consisted of 3 chambers with the dimension of 12 × 20 × 26 cm for the center chamber, and 14 × 20 × 26 cm for side chambers. Both side chambers had plastic cages in the corner to place objects or mice. The experiment consisted of 4 sessions, including habituation in the center camber (10 min), habituation in all chambers (10 min), object/mouse exploration (10 min), and old/novel mouse exploration (10 min). In the third session, a WT stranger mouse was gently confined in a plastic cage, and an inanimate object was placed in the opposite plastic cage. Mice were allowed to freely move and explore, and sniffing and chamber times were measured for the analysis of social interaction. Sniffing was defined as the nose of mouse facing towards and getting close to the target object or mouse. In the last session, the object was replaced with a new WT stranger mouse. The positions of object and stranger 1 were alternated in a random manner to prevent side preference.

### Rotarod test

Mice were placed gently on the rotating rod of the rotarod apparatus (Stoelting), followed by an increase in the speed of the rod from 4 to 40 rpm over 5 min. The experiments were performed for 5 consecutive days, measuring the latency to fall from the rod.

### Morris water maze test

Mice in a circular tank (100 cm diameter) filled with white opaque water (22–24 °C) were trained to find a hidden plat form (10 cm diameter). The learning phase consisted of 3 trials per day for 5 days. For the probe test (1 min), the platform was removed, and the time a mouse spent exploring each quadrant was measured. In the reversal test (3 days), the platform was relocated to the opposite quadrant position. Mouse movements were analyzed using EthoVision XT 10 (Noldus)

### Rewarded alteration T maze

This test was performed as previously described^44^. In brief, underfed mice with the body weight of 80–85% of a normal range were habituated to the T maze arena for 5 min for 2 days, followed by training with 20 μl of 70% milk reward for 10 days.

### Spontaneous alteration T maze

Mice placed in the start point were allowed to freely explore the two arms of the maze. When a mouse enters into one of the two arms, it was brought back to the start point after it spent 5 sec in the arm for the next round, which was repeated 10 times.

### Novel object recognition test

Mice, habituated to an open-field box for 60 min on day 1, were presented with two identical objects for 11 min on day 2. On day 3, one of the objects was replaced with a novel one. Object recognition was measured by the amount of time during which the nose of the mouse pointed the object.

### Contextual fear conditioning and extinction

On day 1, mice were habituated to the shock chamber (Coulbourn Instruments) for 5 min. On day 2, a mouse spent 3 min in the chamber before it received foot shocks (0.8 mA) at 3-, 4-, and 5-min time points, and freezing levels during 3–6 min were quantified. On day 3, mice were re-exposed to the chamber without foot shock for 5 min, and freezing levels during this 5 min were quantified. Freezing behavior was analyzed using FreezeFrame 3 software (Coulbourn Instruments). For contextual fear extinction, mice were exposed to the same shock chamber for 5 min for 10 consecutive days.

### Cued fear conditioning

Mice without habituation spent 3 min in the context-A shock chamber before they received an auditory cue (4 kHz, 75 dB) for 30 sec that co-terminated with 2 sec foot shock (0.8 mA). This conditioning was repeated 3 times with one minute intervals. Twenty four hours later, a mouse was placed in the context B where it spent 3 min before it received the same auditory cue for 3 min. Freezing behavior during this 3 min period was analyzed.

### Contextual discrimination

We subjected mice to a contextual fear conditioning test using a less distinct pair of contexts (A and B) that shared an identical metal grid floor, but had unique wall paper, bottom color, and lighting. In this protocol, fear conditioning took place incrementally over several days in order to investigate the effects of repeated experiences. The experiments were performed in the same condition of time, temperature, and humidity: 2–9 pm, 20–22 °C, and 45%. Each trial was performed after habituation in their cage for 30–60 min and twice a day. During days 1–3 of the experiment, mice were placed in a shock chamber for 180 s, given a single foot-shock of 0.8 mA for 2 s, and taken out after 60 s. On days 4 and 5, for generalization, mice of each genotype were divided into two groups, one visiting chamber A and the other visiting chamber B (one trial/day) for 4 min on day 4, and visiting the unvisited chambers on day 5. Mice did not receive foot-shock during the generalization, and freezing was assessed. During days 6–13, for discrimination training, mice visited the two chambers every day for 8 days, always receiving a foot-shock (0.8 mA for 2 s) at 180 s after being placed in chamber A but not chamber B, followed 1 more min stay. Freezing during the first 3-min exposure in each chamber was used to calculate the daily discrimination ratio.

### Analysis of behavioral results

All the behavioral results were analyzed in a blind manner.

## Additional Information

**How to cite this article**: Park, H. *et al.* Mice lacking the PSD-95–interacting E3 ligase, Dorfin/Rnf19a, display reduced adult neurogenesis, enhanced long-term potentiation, and impaired contextual fear conditioning. *Sci. Rep.* **5**, 16410; doi: 10.1038/srep16410 (2015).

## Supplementary Material

Supplementary Information
