# Supplementary material for: Mice lacking the PSD-95–interacting E3 ligase, Dorfin/Rnf19a, display reduced adult neurogenesis, enhanced long-term potentiation, and impaired contextual fear conditioning
Source: Sci Rep. 2015 Nov 10;5:16410. doi: 10.1038/srep16410 (PMC4639748; doi:10.1038/srep16410)
Supplement: Supplementary Information [file srep16410-s1.doc]

**Supplementary Information**

**Mice lacking the PSD-95–interacting E3 ligase, Dorfin/Rnf19a, display reduced adult neurogenesis, enhanced long-term potentiation, and impaired contextual fear conditioning**

Hanwool Park,^1^ Jinhee Yang,^2^ Ryunhee Kim,^2^ Yan Li,^3^ Yeunkum Lee,^3^ Chungwoo Lee,^2^ Jongil Park,^2^ Dongmin Lee,^4^ Hyun Kim,^4^ and Eunjoon Kim ^2,3^,*

^1^Graduate School of Medical Science and Engineering, Korea Advanced Institute for Science and Technology (KAIST), Daejeon 305-701, Korea; ^2^Department of Biological Sciences, KAIST, Daejeon 305-701, Korea; ^3^Center for Synaptic Brain Dysfunctions, Institute for Basic Science (IBS), Daejeon 305-701, Korea; ^4^Department of Anatomy and Division of Brain Korea 21. Biomedical Science, College of Medicine, Korea University, Seoul 136-704, Korea; *Corresponding author.

| **Fold change** | **Official symbol (HGNC)** | **Site of ubiquitination** | **Official full name (HGNC)** | **Target peptide sequence** | **Known synaptic and behavioral functions** |
| --- | --- | --- | --- | --- | --- |
| -11.7 | Cxx1a | 85 | CAAX box 1A | ALQWVIPYIK*IDSPLLNDYNGFLNEMK | Unknown |
| -8.5 | Atp6v1a | 438 | V-type proton ATPase catalytic subunit A | EGSVSIVGAVSPPGGDFSDPVTSATLGIVQVFWGLDKK*LAQR | Enriched in the PSD^1, 2^ |
| -5.7 | Plxna1; Plxna4 | 1295; 1289 | Plexin A1; Plexin A4 | VALECK*EAFAELQTDIHELTSDLDGAGIPFLDYR | Enriched in the PSD ^3^  PLXNA1 KO mouse show normal spine density in DG ^4^ |
| -4 | Npepps | 80 | Puromycin-sensitive aminopeptidase | LPAEVSPINYSLCLKPDLLDFTFEGK*LEAAAQVR | Enriched in the PSD ^3^ |
| -3.8 | H2afz | 16 | H2A histone family, member Z | TK*AVSR | Negative regulator of contextual fear memory ^5^ |
| -3.8 | Gnpda1 | 208 | glucosamine-6-phosphate deaminase 1 | FFDGDLAKVPTMALTVGVGTVMDAKEVMILITGAHK* | Unknown |
| -3.8 | Rab11b | 145 | RAB11B, member RAS oncogene family | AFAEK*NNLSFIETSALDSTNVEEAFKNILTEIYR | Translocates GluR1 into spines during LTP ^6, 7, 8, 9, 10^ CREB/memory-induced target in C. elegans ^11^ |
| -3.5 | Gltp | 46 | Glycolipid transfer protein | QIETGPFLEAVAHLPPFFDCLGSPVFTPIK*ADISGNITK | Unknown |
| -3.3 | Gnai1 | 345 | guanine nucleotide binding protein (G protein), alpha inhibiting activity polypeptide 1 | NVQFVFDAVTDVIIK*NNLKDCGLF | Enriched in the PSD ^3^  GNAI1 KO mice show enhanced CA1 LTP, normal spatial memory, reduced contextual fear memory ^12^ |
| -3.3 | Rnmt | 179 | RNA (guanine-7-) methyltransferase | ILEK*VR | Unknown |
| -3.2 | Psmb2 | 62 | proteasome (prosome, macropain) subunit, beta type, 2 | ILLLCVGEAGDTVQFAEYIQK*NVQLYK | Unknown |
| -3.2 | Ppp1ca; Ppp1cc | 60; 60 | protein phosphatase 1, catalytic subunit, alpha/gamma isozyme | EIFLSQPILLELEAPLK*ICGDIHGQYYDLLR | Enriched in the PSD ^1, 3^  Required for induction and expression of LTD ^13, 14, 15^  Inhibited during LTP ^15, 16^  Suppressor of learning and memory ^17, 18, 19, 20^ |
| -3 | Ppp1cb | 59 | protein phosphatase 1, catalytic subunit, beta isozyme | EIFLSQPILLELEAPLK*ICGDIHGQYTDLLR | Enriched in the PSD ^1, 3^  Required for induction and expression of LTD ^13, 14, 15^  Inhibited during LTP ^15, 16^  Suppressor of learning and memory ^17, 18, 19, 20^ |
| -2.9 | Ywhah  (Also known as 14-3-3η) | 217 | tyrosine 3-monooxygenase/tryptophan 5-monooxygenase activation protein, eta | QAFDDAIAELDTLNEDSYK*DSTLIMQLLR | Enriched in the PSD ^1, 3, 21^  Functional KO mice show reduced CA1 LTP, NMDA/AMPA ratio, and contextual fear conditioning ^22^  Contextual fear conditioning increases expression levels of 14-3-3η ^23^ |
| -2.8 | Ywhaz  (Also known as 14-3-3ζ) | 103 | tyrosine 3-monooxygenase/tryptophan 5-monooxygenase activation protein, zeta | DICNDVLSLLEK*FLIPNASQPESK | Enriched in the PSD ^1, 2, 3, 24^  Functional KO mice show reduced CA1 LTP, NMDA/AMPA ratio, and contextual fear conditioning ^22^ |
| -2.8 | Fasn | 197 | Fatty acid synthase | LLLPEDPLISGLLNSQALK*ACVDTALENLSTLK | Unknown |
| -2.7 | Faah | 295 | Fatty acid amide hydrolase | DVDSLALCMK*ALLCEDLFR | Enriched in the PSD ^3^  FAAH inhibitor inhibits LTP and impairs novel object recognition, spontaneous alternation, and spatial memory ^25^  FAAH inhibitor facilitates auditory fear extinction and aversive inhibitory avoidance ^26, 27^ |
| -2.6 | Gsn | 97 | Gelsolin | VEKFDLVPVPPNLYGDFFTGDAYVILK*TVQLR | Enriched in the PSD ^3, 24^  Stabilization of actin filaments during synaptic plasticity ^28, 29^  Gelsolin KO mice show normal LTD of NMDAR EPSCs in CA1 ^30^  Promotes filopodium-spine transition induced by syndecan-2 ^31^  Upregulated in the DG during spatial memory consolidation ^32^ |
| -2.6 | Nfm | 460 | Neurofilament medium polypeptide | FVEEIIEETK*VEDEKSEMEETLTAIAEELAASAK | Enriched in the PSD ^24^ |
| -2.6 | Chn1 | 52 | chimerin 1 | SSVTIWQPLK*LFAYSQLTSLVR | KO mice show increased contextual fear learning but normal cued fear learning ^33^ |
| -2.6 | Psmd7 | 219 | proteasome (prosome, macropain) 26S subunit, non-ATPase, 7 | VASGK*LPINHQIIYQLQDVFNLLPDASLQEFVK | Unknown |
| -2.5 | Fam177a | 115 | family with sequence similarity 177, member A | AATSTLSVCDFLGEK*IASVLGISTPK | Unknown |

***Supplementary Table 1.*** List of proteins whose ubiquitination levels were decreased by > 2.5 folds in the *Dorfin*^–/–^ hippocampus relative to WT controls. Their known associations with synaptic plasticity and learning and memory behavior are also indicated. HGNC, HUGO gene nomenclature committee.

**
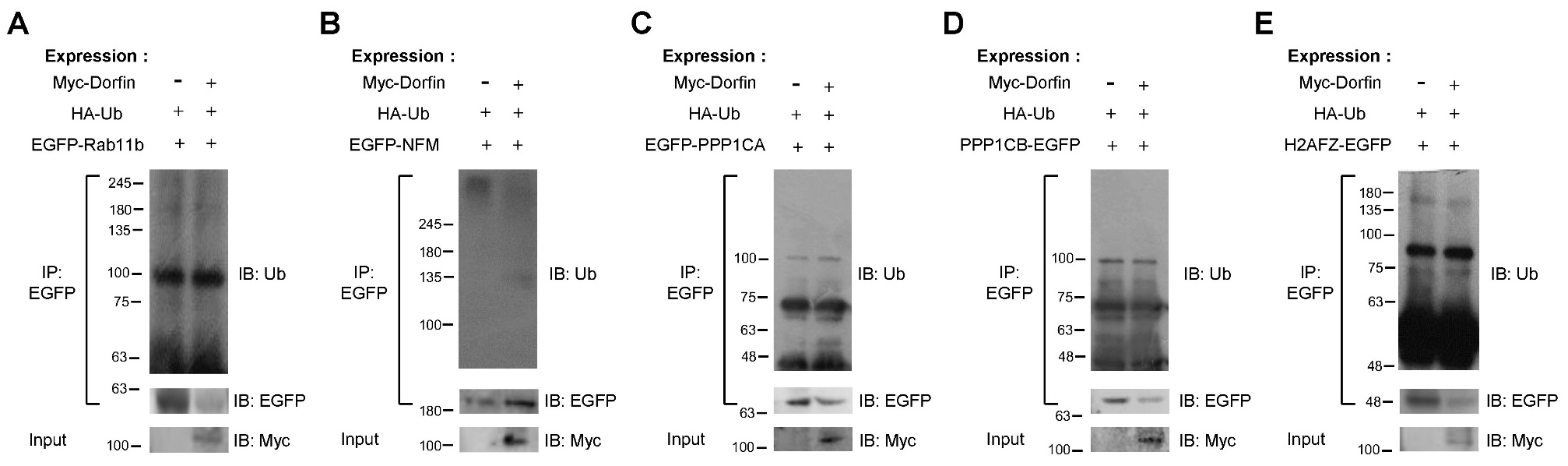
**

**
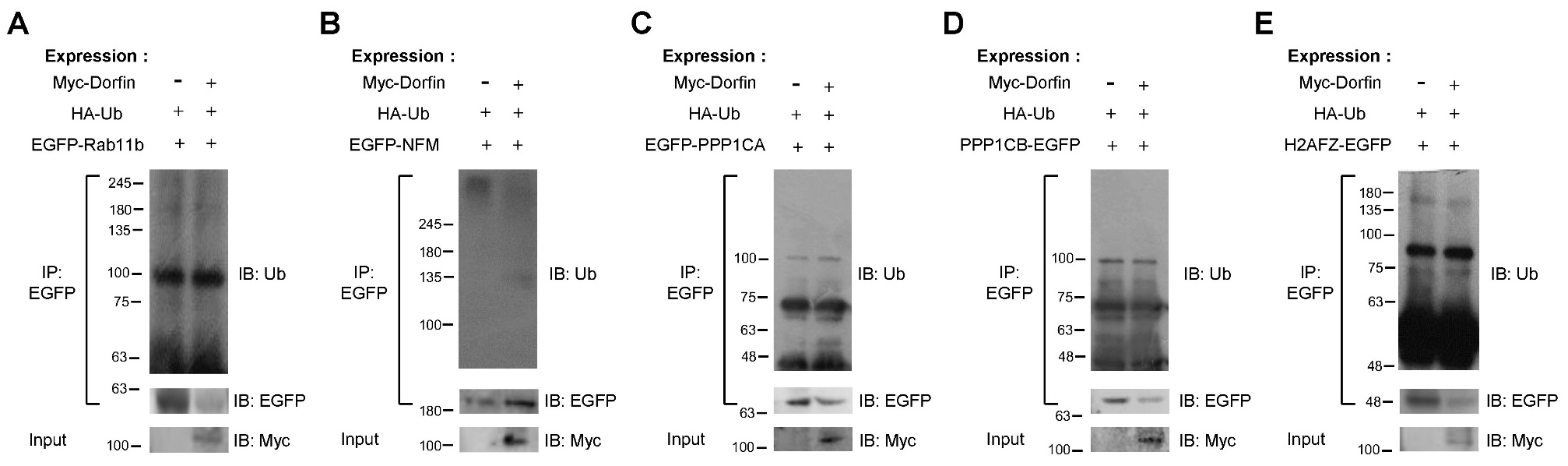
**

**Supplementary Figure 1. Five Dorfin-associated proteins are not ubiquitinated by Dorfin in heterologous cells.**

(**A-E**) EGFP-tagged Dorfin associated proteins (Rab11b, neurofilament M [NFM], the α and β subunits of PP1 phosphatase [PPP1CA and PPP1CB], and H2AFZ) were coexpressed with Myc-Dorfin and HA-ubiquitin (HA-Ub) in HEK293T cells, followed by immunoprecipitation with EGFP antibodies and immunoblotting with the indicated antibodies.

**References**

1. Dosemeci A*, et al.* Composition of the synaptic PSD-95 complex. *Mol Cell Proteomics* **6**, 1749-1760 (2007).

2. Moron JA*, et al.* Morphine administration alters the profile of hippocampal postsynaptic density-associated proteins: a proteomics study focusing on endocytic proteins. *Mol Cell Proteomics* **6**, 29-42 (2007).

3. Bayes A*, et al.* Characterization of the proteome, diseases and evolution of the human postsynaptic density. *Nat Neurosci* **14**, 19-21 (2011).

4. Duan Y*, et al.* Semaphorin 5A inhibits synaptogenesis in early postnatal- and adult-born hippocampal dentate granule cells. *Elife* **3**, (2014).

5. Zovkic IB, Paulukaitis BS, Day JJ, Etikala DM, Sweatt JD. Histone H2A.Z subunit exchange controls consolidation of recent and remote memory. *Nature* **515**, 582-586 (2014).

6. Correia SS*, et al.* Motor protein-dependent transport of AMPA receptors into spines during long-term potentiation. *Nat Neurosci* **11**, 457-466 (2008).

7. Keith DJ*, et al.* Palmitoylation of A-kinase anchoring protein 79/150 regulates dendritic endosomal targeting and synaptic plasticity mechanisms. *J Neurosci* **32**, 7119-7136 (2012).

8. Wang Z*, et al.* Myosin Vb mobilizes recycling endosomes and AMPA receptors for postsynaptic plasticity. *Cell* **135**, 535-548 (2008).

9. Petrini EM*, et al.* Endocytic trafficking and recycling maintain a pool of mobile surface AMPA receptors required for synaptic potentiation. *Neuron* **63**, 92-105 (2009).

10. Park M, Penick EC, Edwards JG, Kauer JA, Ehlers MD. Recycling endosomes supply AMPA receptors for LTP. *Science* **305**, 1972-1975 (2004).

11. Lakhina V*, et al.* Genome-wide functional analysis of CREB/long-term memory-dependent transcription reveals distinct basal and memory gene expression programs. *Neuron* **85**, 330-345 (2015).

12. Pineda VV*, et al.* Removal of G(ialpha1) constraints on adenylyl cyclase in the hippocampus enhances LTP and impairs memory formation. *Neuron* **41**, 153-163 (2004).

13. Mulkey RM, Herron CE, Malenka RC. An essential role for protein phosphatases in hippocampal long-term depression. *Science* **261**, 1051-1055 (1993).

14. Thiels E, Norman ED, Barrionuevo G, Klann E. Transient and persistent increases in protein phosphatase activity during long-term depression in the adult hippocampus in vivo. *Neuroscience* **86**, 1023-1029 (1998).

15. Jouvenceau A*, et al.* Partial inhibition of PP1 alters bidirectional synaptic plasticity in the hippocampus. *Eur J Neurosci* **24**, 564-572 (2006).

16. Blitzer RD*, et al.* Gating of CaMKII by cAMP-regulated protein phosphatase activity during LTP. *Science* **280**, 1940-1942 (1998).

17. Genoux D*, et al.* Protein phosphatase 1 is a molecular constraint on learning and memory. *Nature* **418**, 970-975 (2002).

18. Koshibu K*, et al.* Protein phosphatase 1 regulates the histone code for long-term memory. *J Neurosci* **29**, 13079-13089 (2009).

19. Koshibu K, Graff J, Mansuy IM. Nuclear protein phosphatase-1: an epigenetic regulator of fear memory and amygdala long-term potentiation. *Neuroscience* **173**, 30-36 (2011).

20. Graff J, Koshibu K, Jouvenceau A, Dutar P, Mansuy IM. Protein phosphatase 1-dependent transcriptional programs for long-term memory and plasticity. *Learn Mem* **17**, 355-363 (2010).

21. Yoshimura Y*, et al.* Molecular constituents of the postsynaptic density fraction revealed by proteomic analysis using multidimensional liquid chromatography-tandem mass spectrometry. *J Neurochem* **88**, 759-768 (2004).

22. Qiao H, Foote M, Graham K, Wu Y, Zhou Y. 14-3-3 proteins are required for hippocampal long-term potentiation and associative learning and memory. *J Neurosci* **34**, 4801-4808 (2014).

23. Kida S*, et al.* CREB required for the stability of new and reactivated fear memories. *Nat Neurosci* **5**, 348-355 (2002).

24. Peng J*, et al.* Semiquantitative proteomic analysis of rat forebrain postsynaptic density fractions by mass spectrometry. *J Biol Chem* **279**, 21003-21011 (2004).

25. Basavarajappa BS, Nagre NN, Xie S, Subbanna S. Elevation of endogenous anandamide impairs LTP, learning, and memory through CB1 receptor signaling in mice. *Hippocampus* **24**, 808-818 (2014).

26. Gunduz-Cinar O*, et al.* Convergent translational evidence of a role for anandamide in amygdala-mediated fear extinction, threat processing and stress-reactivity. *Mol Psychiatry* **18**, 813-823 (2013).

27. Morena M*, et al.* Endogenous cannabinoid release within prefrontal-limbic pathways affects memory consolidation of emotional training. *Proc Natl Acad Sci U S A* **111**, 18333-18338 (2014).

28. Disanza A*, et al.* Eps8 controls actin-based motility by capping the barbed ends of actin filaments. *Nat Cell Biol* **6**, 1180-1188 (2004).

29. Star EN, Kwiatkowski DJ, Murthy VN. Rapid turnover of actin in dendritic spines and its regulation by activity. *Nat Neurosci* **5**, 239-246 (2002).

30. Morishita W, Marie H, Malenka RC. Distinct triggering and expression mechanisms underlie LTD of AMPA and NMDA synaptic responses. *Nat Neurosci* **8**, 1043-1050 (2005).

31. Hu HT, Hsueh YP. Calcium influx and postsynaptic proteins coordinate the dendritic filopodium-spine transition. *Dev Neurobiol* **74**, 1011-1029 (2014).

32. Monopoli MP*, et al.* Temporal proteomic profile of memory consolidation in the rat hippocampal dentate gyrus. *Proteomics* **11**, 4189-4201 (2011).

33. Iwata R*, et al.* RacGAP alpha2-chimaerin function in development adjusts cognitive ability in adulthood. *Cell Rep* **8**, 1257-1264 (2014).
